# Optimizing Urban Traffic Efficiency and Safety via V2X: A Simulation Study Using the MOSAIC Platform †

**DOI:** 10.3390/s25175418

**Published:** 2025-09-02

**Authors:** Sebastian-Ioan Alupoaei, Constantin-Florin Caruntu

**Affiliations:** Department of Automatic Control and Applied Informatics, “Gheorghe Asachi” Technical University of Iasi, 700050 Iasi, Romania; sebastian-ioan.alupoaei@student.tuiasi.ro

**Keywords:** vehicle-to-everything (V2X), vehicle-to-vehicle (V2V), vehicle-to-infrastructure (V2I), intelligent transportation systems (ITS), traffic simulation, urban mobility, Eclipse MOSAIC, Simulation of Urban Mobility (SUMO), traffic congestion mitigation, cooperative rerouting, smart cities, multi-incident traffic management

## Abstract

Urban growth and rising vehicle usage have intensified congestion, accidents, and environmental impact, exposing the limitations of traditional traffic management systems. This study introduces a dual-incident simulation framework to investigate the potential of Vehicle-to-Everything (V2X) technologies in enhancing urban mobility. Using the Eclipse MOSAIC platform integrated with SUMO, a realistic network in Iași, Romania, was modeled under single- and dual-incident scenarios with three V2X penetration levels: 0%, 50%, and 100%. Unlike prior works that focus on single-incident cases or assume full penetration, our approach evaluates cascading disruptions under partial adoption, providing a more realistic transition path for mid-sized European cities. Key performance indicators, i.e., average speed, vehicle density, time loss, and waiting time, were calculated using mathematically defined formulas and validated across multiple simulation runs. Results demonstrate that full V2X deployment reduces average time loss by 18% and peak density by more than 70% compared to baseline conditions, while partial adoption delivers measurable yet limited benefits. The dual-incident scenario shows that V2X-enabled rerouting significantly mitigates cascading congestion effects. These contributions advance the state of the art by bridging microscopic vehicle dynamics with network-level communication modeling, offering quantitative insights for phased V2X implementation and the design of resilient, sustainable intelligent transportation systems.

## 1. Introduction

Accelerating urbanization and increasing reliance on cars have created a multitude of complex and major challenges for traditional transportation systems, requiring a shift towards more efficient and sustainable solutions. Issues of urban traffic jams, accidents, and pollution [1] have emerged as significant barriers to efficient and sustainable mobility, compounding the problems with each additional vehicle on city streets [2]. This trend results in growing traffic delays, higher economic costs, and increased public frustration, reaching alarming levels [3].

Vehicle-to-everything (V2X) communication, which facilitates real-time data exchange among vehicles, infrastructure, and other road users, represents a key enabler for next-generation intelligent transportation systems (ITSs). Unlike conventional traffic management approaches that depend heavily on static infrastructure such as traffic lights and fixed road sensors, V2X establishes a decentralized, adaptive network of connected vehicles [4]. Through direct vehicle-to-vehicle and vehicle-to-infrastructure communication, V2X systems can dynamically share information on traffic conditions, potential hazards, and optimal routing strategies. This real-time, cooperative exchange reduces reliance on fixed control mechanisms and enables seamless operation even in environments lacking conventional infrastructure. As a result, V2X-based solutions offer a scalable and versatile framework for mitigating traffic congestion and enhancing mobility efficiency across diverse deployment scenarios [5].

One solution proposed by [6,7] is to use the Adaptive Route Change (ARC) application to improve traffic flow in cities by cutting down on fuel use and easing congestion. It works by using Vehicle-to-Infrastructure (V2I) communications to give drivers real-time suggestions for alternative routes whenever traffic jams are detected. Nevertheless, in [8], a cost-efficient and eco-friendly approach is suggested for real-time estimation of traffic density and signal management using live video streams from current surveillance cameras situated at traffic intersections. The algorithm adjusts traffic signals according to vehicle density to alleviate congestion and minimize waiting times, all the while acquiring valuable data for road planning and analysis purposes. Another method for rerouting vehicles presented in [9] involves the following steps: first, each Roadside Unit (RSU) obtains the current position of the vehicle as the source position and identifies the last edge of the vehicle’s route within its coverage as the destination. The vehicle’s route is then divided, and the *k* Shortest Paths algorithm is applied. Each RSU determines the *k* possible routes. After selecting these routes, traffic is balanced using the Boltzmann probability distribution. Finally, the remainder of the original route is appended to the newly calculated route and sent to the vehicle. The research conducted in [10] aimed to reduce congestion in urban areas by using a multi-variable model to estimate the appropriate charge. The data for this study were collected from surveys conducted in primary congested zones. The model was evaluated using Fisher’s multiple regression analysis and the coefficient of determination, which indicated that the model is statistically significant.

In response to these critical challenges, this paper presents a comprehensive investigation into the potential of ITSs [11] to transform urban mobility through advanced V2X-enabled solutions. Utilizing the MOSAIC platform in conjunction with the Simulation of Urban Mobility (SUMO) framework [12,13], this study conducts a detailed analysis of traffic dynamics in the city of Iași, with a focus on high-impact corridors such as Tudor Vladimirescu Boulevard, Chimiei Boulevard, and Palat Street. Key traffic performance indicators, including vehicle density and average speed, are systematically evaluated to develop optimal strategies for enhancing efficiency and safety in urban transport networks. By integrating large-scale simulation with data-driven modeling, this research generates actionable insights for reducing congestion, optimizing traffic flow, and fostering sustainable mobility [14,15,16]. Beyond addressing current bottlenecks, the findings provide a robust foundation for the development of future ITS frameworks, demonstrating how V2X-based architectures can drive innovative, adaptive, and environmentally sustainable solutions for urban transportation systems.

This work extends the research in [17], which simulated a single-incident scenario using the Eclipse MOSAIC platform in a representative area of Iași, Romania. The present study introduces a dual-incident scenario and examines its combined impact on traffic congestion, average waiting time, and vehicle rerouting. It integrates advanced communication modeling, expands the geographic scope to include Calea Chișinăului, and compares performance across varying levels of V2X penetration (0%, 50%, and 100%). Additionally, a statistical evaluation of time loss and waiting time enhances the robustness of the results and informs future urban traffic management strategies.

While many studies explore V2X-based intelligent transportation systems, most focus on single-intersection or single-incident scenarios and often assume full V2X penetration. There is limited research addressing network-wide cascading disruptions under varying levels of technology adoption, especially in mid-sized European urban contexts. This study addresses these gaps by:

- Developing a dual-incident simulation framework that evaluates both single and multiple disruptions in a realistic urban road network.
- Assessing the effect of different V2X penetration levels (0%, 50%, and 100%) on key performance indicators, validated through statistical methods.
- Integrating Eclipse MOSAIC and SUMO to combine microscopic traffic dynamics with network-level communication modeling.
- Providing actionable insights for municipal traffic planners, highlighting thresholds for partial adoption and strategies to enhance resilience under multiple-incident conditions.

These contributions extend current V2X research by bridging the gap between simulation-based evaluations and practical urban deployment strategies.

The paper is structured as follows: Section 2 presents the impact of the current work with respect to the state-of-the-art on this topic. Section 3 presents the methods employed for modeling traffic dynamics, V2X communication, and performance indicators. Section 4 provides an overview of the Eclipse MOSAIC simulator, the simulation scenarios used in this study, and explores the communication strategies used in the simulation, while Section 5 presents the experimental results. Section 6 presents an in-depth analysis of the obtained results and discusses them in correlation with a base scenario. Finally, Section 7 summarizes the conclusions drawn from this research and highlights the implications for future intelligent transportation systems.

## 2. Impact and Related Work

Urban mobility systems are undergoing a paradigm shift driven by rapid urbanization, increased vehicle ownership, and growing demands for sustainable transport infrastructure. Traditional centralized traffic management systems are often unable to adapt to real-time disruptions, resulting in persistent congestion, increased travel times, and significant environmental impact due to elevated CO_2_ emissions and fuel consumption. Studies estimate that urban traffic inefficiencies account for billions in annual economic losses worldwide and up to 25% of unnecessary emissions in densely populated areas [18,19].

To address these challenges, intelligent transportation systems (ITSs) have emerged as an interdisciplinary solution integrating Vehicle-to-Everything (V2X) communication, advanced sensing, and AI-driven control for adaptive traffic optimization. V2X encompasses Vehicle-to-Vehicle (V2V), Vehicle-to-Infrastructure (V2I), and Vehicle-to-Network (V2N) paradigms, enabling cooperative and predictive decision-making in complex traffic environments. Recent studies show that cooperative V2X deployment can reduce collision rates by up to 81% and cut average travel times by more than 30% under mixed traffic conditions [20].

Emerging Cooperative-ITS (C-ITS) platforms leverage hybrid communication models combining IEEE 802.11p/ITS-G5, Cellular V2X (C-V2X), and edge/fog computing architectures to enable reliable, low-latency message exchange. Zadobrischi et al. [19] demonstrated the effectiveness of C-V2X and Visible Light Communication (VLC) in dense urban environments, achieving sub-10 ms latency and <5% packet loss while maintaining robust vehicular positioning and routing. Similarly, Wang et al. [21] quantified the role of C-V2X in mixed traffic scenarios, showing that hybrid V2V/V2I networks significantly improve rerouting efficiency during disruptions.

Simulation-based validation remains essential for assessing ITS strategies before deployment. Multi-agent reinforcement learning approaches have recently emerged as a dominant paradigm. Liang et al. [22] applied deep reinforcement learning (DRL) for adaptive traffic signal control, minimizing cumulative waiting times using real-time vehicular data [22]. Wu et al. [23] analyzed DRL reward structures for intersection optimization, highlighting policy sensitivity to varying traffic demands. Li et al. [18] introduced a federated DRL framework for distributed multi-intersection coordination, achieving scalable, privacy-preserving traffic management Other studies emphasize IoT-enhanced sensing and predictive analytics as key enablers of dynamic traffic management, integrating real-time sensor data with AI-based control [20,24,25,26,27].

Beyond simulation frameworks such as SUMO [13], Veins [28], and MOSAIC [12], a rich body of research has explored mathematical and algorithmic approaches for traffic flow optimization. Microscopic and agent-based modeling has been applied to capture complex V2V, V2P, and V2X interactions in heterogeneous traffic [29], providing insights into emergent behaviors that traditional simulation tools cannot fully reproduce. Recent surveys highlight the diversity of agent-based traffic simulators and their ability to support realistic mobility scenarios with decentralized decision-making [30]. In parallel, metaheuristic optimization methods such as genetic algorithms (GAs) and particle swarm optimization (PSO) have been successfully applied to traffic control and network design problems, offering scalable solutions for congestion mitigation and reconfigurable network planning [31,32]. For example, a multi-agent hybrid clustering-assisted GA for the evolutionary synthesis of multi-layer road networks is proposed in [33], demonstrating the potential of algorithmic approaches to design high-capacity and resilient infrastructures. While our study focuses on the MOSAIC–SUMO platform to evaluate V2X adoption under single- and dual-incident scenarios, these complementary directions—agent-based modeling and simulation-based optimization—remain highly relevant and will be considered in future research extensions.

Despite these advances, gaps remain. Many works evaluate single-intersection or single-incident scenarios, neglecting the multi-incident resilience required in real urban networks. Additionally, the impact of varying V2X penetration levels on traffic flow under disruptive conditions is underexplored, with most studies assuming full deployment. Furthermore, performance metrics often focus on average speed or throughput while overlooking temporal indicators such as waiting time and time loss per vehicle, which are critical for municipal decision-making [34].

The following table (Table 1) provides a comparative overview between this study and relevant works in the field of intelligent transportation systems (ITS), highlighting simulation environments, scenarios, key metrics, and communication technologies:

The present study addresses these gaps by proposing a multi-scenario, multi-incident simulation framework using the Eclipse MOSAIC platform coupled with SUMO. It evaluates the effects of varying V2X penetration levels (0%, 50%, and 100%) under both single- and dual-accident conditions in a real-world-inspired urban network in Iași, Romania. Using Cellular Geobroadcast as the communication backbone, the framework integrates realistic message propagation, rerouting strategies, and mixed traffic dynamics. Key performance indicators, including average speed, waiting time, and time loss, are statistically validated across multiple runs to ensure reproducibility.

By bridging the gap between microscopic traffic simulation and network-level communication modeling, this work extends the state-of-the-art in V2X-enabled ITS research. Unlike prior single-intersection DRL or isolated C-V2X studies, it models network-wide cascading disruptions and evaluates cooperative rerouting under partial and full technology adoption scenarios. These insights directly support the design of digital twins for smart cities, enabling municipalities to assess the robustness and scalability of ITS solutions under realistic perturbations and transitional technology rollouts.

## 3. Methods

The methodological framework integrates microscopic traffic modeling, vehicular communication, and cooperative rerouting within the Eclipse MOSAIC–SUMO environment. Vehicle dynamics are represented through car-following and lane-changing models, while incidents generate V2X messages that inform rerouting decisions for equipped vehicles. This workflow links mobility, communication, and application layers, producing realistic mixed-traffic responses to disruptive events. Performance is assessed using standard indicators such as average speed, density, travel time, time loss, waiting time, and queue length, each derived from simulation outputs and formalized for reproducible comparisons.

### 3.1. Traffic Flow Models

The traffic flow simulations rely on microscopic vehicle models implemented within SUMO and orchestrated through MOSAIC. Each vehicle is represented as an autonomous agent whose state is defined by position, velocity, and acceleration. The vehicle dynamics follow the Intelligent Driver Model (IDM) for longitudinal motion, expressed as a set of nonlinear differential equations that capture acceleration based on desired speed, headway distance, and safe time gap. For lane-changing behavior, the MOBIL model is applied, which evaluates incentive and safety criteria to determine when a maneuver is feasible. Together, these models provide a realistic description of car-following and lane-changing interactions in heterogeneous traffic. By integrating these microscopic models into the MOSAIC framework, we ensure that communication events (such as the reception of a DENM) directly influence the trajectory decisions of individual vehicles, thereby linking mathematical vehicle dynamics with cooperative communication strategies.

#### 3.1.1. Intelligent Driver Model (IDM)

The longitudinal dynamics of a vehicle *i* are given by the Intelligent Driver Model (IDM) as follows:

(1) $${\dot{v}}_{i}=a\left[1-{\left(\frac{v_{i}}{v_{0}}\right)}^{\delta }-{\left(\frac{s^{*}\left(v_{i},\Delta v_{i}\right)}{s_{i}}\right)}^{2}\right],$$

where $v_{i}$ is the velocity of vehicle *i*, $s_{i}$ is the net distance to the leading vehicle, and $\Delta v_{i}=v_{i}-v_{\ell (i)}$ is the relative velocity with respect to the leader. The desired dynamic gap is

(2) $$s^{*}\left(v_{i},\Delta v_{i}\right)=s_{0}+v_{i}T+\frac{v_{i}\;\Delta v_{i}}{2\sqrt{ab}}.$$

The model parameters are $v_{0}$ (desired speed), *a* (maximum acceleration), *b* (comfortable deceleration), *T* (desired time headway), $s_{0}$ (minimum gap), and δ (acceleration exponent, typically δ=4). The vehicle position evolves as ${\dot{x}}_{i}=v_{i}$.

#### 3.1.2. MOBIL Lane-Changing Model

Lane-changing decisions are governed by the MOBIL model, which combines an incentive criterion and a safety criterion. The incentive criterion compares the acceleration gains of the ego vehicle with the losses imposed on neighboring vehicles:

(3) $$\left(a_{i}^{\mathrm{new}}-a_{i}^{\mathrm{old}}\right)+p\left[\left(a_{f}^{\mathrm{new}}-a_{f}^{\mathrm{old}}\right)+\left(a_{\tilde{f}}^{\mathrm{new}}-a_{\tilde{f}}^{\mathrm{old}}\right)\right]>\Delta a_{\mathrm{th}}+b_{\mathrm{bias}},$$

where $a_{i}$ denotes the acceleration of the ego vehicle, $a_{f}$ the follower in the current lane, and $a_{\tilde{f}}$ the follower in the target lane, each computed with the chosen car-following model (e.g., IDM). Superscripts “old” and “new” indicate values before and after a hypothetical lane change.

The parameters are *p* (politeness factor), $\Delta a_{\mathrm{th}}$ (minimum advantage threshold), and $b_{\mathrm{bias}}$ (lane bias term).

The safety criterion ensures that the deceleration of followers remains acceptable:

(4) $$a_{\tilde{f}}^{\mathrm{new}}\geq -b_{\mathrm{safe}}\;\mathrm{and}\;a_{f}^{\mathrm{new}}\geq -b_{\mathrm{safe}},$$

where $b_{\mathrm{safe}}$ is the maximum safe braking deceleration. A lane change is performed only if both criteria are satisfied.

### 3.2. V2X Communication Modeling

Communication between vehicles and infrastructure is represented through the MOSAIC communication federate, which supports both IEEE 802.11p (Dedicated Short-Range Communications) and LTE-based vehicular networking. In the simulated scenarios, disruptive events trigger the generation of Decentralized Environmental Notification Messages (DENMs), which are broadcast to nearby vehicles within the communication range. Message propagation accounts for latency, packet loss, and channel capacity constraints to reflect realistic network behavior. Equipped vehicles receiving DENMs update their routing decisions based on the latest incident information, while non-equipped vehicles remain unaffected. This approach enables a differentiated response within mixed-traffic conditions, where the proportion of V2X-enabled vehicles directly influences overall network performance. By integrating communication parameters into the mobility layer, the model captures both the opportunities and limitations of V2X-assisted traffic management.

### 3.3. Simulation Workflow and Algorithmic Logic

**Figure 1:**
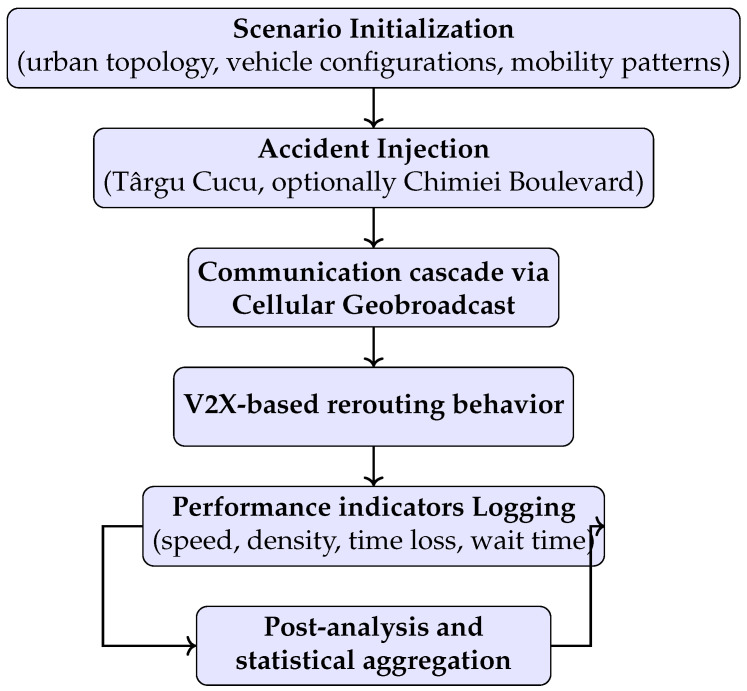
Simulation workflow and architecture.

To evaluate the influence of V2X communication on urban traffic dynamics, a structured simulation workflow was implemented in the Eclipse MOSAIC framework (Figure 1). The architecture comprises six stages designed to replicate realistic urban scenarios with incident-induced disruptions. It begins with scenario initialization, defining the network topology, vehicle configurations, and mobility patterns, followed by the introduction of an accident event to simulate unexpected disturbances. This triggers vehicle-to-vehicle and vehicle-to-infrastructure messaging via cellular geobroadcast, enabling real-time rerouting. Throughout the simulation, key performance indicators, i.e., average speed, vehicle density, time loss, and waiting time, are continuously monitored. A post-processing stage aggregates and analyzes these metrics to assess system performance across different V2X penetration levels. This modular workflow provides a consistent framework for simulating complex traffic behavior and optimizing rerouting strategies to mitigate the impact of incidents on urban mobility.

The workflow consists of five steps:

1. **Initialization (stage 1):** Load network topology, vehicle configurations, and demand patterns.
2. **Accident injection (stage 2):** Introduce incident(s) as static obstacles with defined coordinates and duration.
3. **Message dissemination (stage 3):** Broadcast DENMs to affected vehicles.
4. **Adaptive rerouting (stage 4):** Vehicles compute alternative paths based on received messages.
5. **Performance logging (stage 5 and 6):** Collect performance indicators for post-analysis.

The process is illustrated in Figure 1, with additional pseudo-code provided by Algorithm 1:
**Algorithm 1:** Accident detection and rerouting procedure.  1:**if** accident_detected **then**  2:   generate DENM (location, severity, duration)  3:   broadcast DENM to vehicles within radius *R*  4:   **for** each vehicle receiving DENM **do**  5:     **if** current_route intersects blocked_area **then**  6:        compute alternative_route via cost-based algorithm  7:        update vehicle trajectory  8:     **end if**  9:   **end for**10:**end if**

### 3.4. Performance Metrics

To quantitatively evaluate the scenarios, we defined a set of *performance indicators* that capture efficiency, resilience, and user experience:

**Average speed**

(5) $$\bar{v}=\frac{1}{N_{\mathrm{veh}}}\sum_{i=1}^{N_{\mathrm{veh}}}v_{i},$$

where $N_{\mathrm{veh}}$ is the number of vehicles.

**Traffic density**

(6) $$\rho =\frac{N_{\mathrm{veh}}}{L},$$

where *L* is the segment length.

**Time loss**

(7) $$T_{\mathrm{loss}}=\frac{1}{N_{\mathrm{veh}}}\sum_{i=1}^{N_{\mathrm{veh}}}\left(t_{i}-t_{i,\mathrm{ideal}}\right),$$

where $t_{i}$ is the actual travel time and $t_{i,\mathrm{ideal}}$ is the expected travel time at maximum allowed speed.

**Waiting time**

(8) $$T_{\mathrm{wait}}=\frac{1}{N_{\mathrm{veh}}}\sum_{i=1}^{N_{\mathrm{veh}}}t_{i,\mathrm{stop}},$$

where $t_{i,\mathrm{stop}}$ is the total time vehicle *i* remains at speed below 0.1 m/s.

**Road Segment Exit Time**

The *road segment exit time* $T_{exit}$ represents the duration required for all vehicles to leave a predefined road segment after entering it. It is computed as the difference between the exit time of the last vehicle and the entry time of the first vehicle into the segment:

$$T_{exit}=\max_{i\in \{1,\ldots ,N_{\mathrm{veh\_seg}}\}}\;t_{i}^{\mathrm{exit}}\;-\;\min_{i\in \{1,\ldots ,N_{\mathrm{veh\_seg}}\}}\;t_{i}^{\mathrm{entry}}$$

where:

- $t_{i}^{\mathrm{entry}}$ is the time at which vehicle *i* enters the road segment;
- $t_{i}^{\mathrm{exit}}$ is the time at which vehicle *i* leaves the segment;
- $N_{\mathrm{veh\_seg}}$ is the total number of vehicles passing through the segment.

This indicator quantifies how quickly traffic is able to clear a congested road section under different V2X penetration rates. A lower $T_{exit}$ value indicates faster recovery from congestion and improved traffic throughput.

Collectively, these KPIs provide a multi-dimensional assessment of traffic performance, allowing us to compare scenarios in terms of both mobility and resilience. All scenarios were executed five times with identical parameters to ensure statistical robustness. Mean values and standard deviations are reported in Section 6.

## 4. The MOSAIC Simulator and Simulation Scenarios

This section illustrates the Eclipse MOSAIC simulator [12], a powerful tool for modeling and analyzing urban transport systems. An overview of the simulator and the simulation scenarios designed for this research will be presented, including the selected urban areas, vehicle configurations, communication protocols, and accident notification methodology used to test and optimize ITS strategies.

### 4.1. MOSAIC Simulator

Eclipse MOSAIC features robust traffic simulation functionalities, providing the possibility to simulate complex and large-scale traffic scenarios. This is achieved by fully integrating the Eclipse Simulation of Urban Mobility (SUMO) simulator [13], a renowned traffic simulation tool, which is directly coupled with MOSAIC via the Traffic Control Interface (TraCI) [38]. This integration allows the simulation of realistic traffic dynamics, including V2V and V2I interactions.

The traffic simulation capabilities of Eclipse MOSAIC are particularly well suited for virtual testing and evaluation of mobility and connected and automated driving solutions. The platform provides a comprehensive and realistic simulation environment, allowing developers and researchers to test and optimize their applications in a controlled and repeatable manner. With Eclipse MOSAIC, users can simulate a wide range of traffic scenarios, including urban and freeway environments, intersections, and roundabouts. The platform also supports the simulation of different types of vehicles, including cars, trucks, buses, and pedestrians. In addition, MOSAIC allows the integration of real-world data, such as traffic signal control and traffic management strategies, to create a highly realistic simulation environment.

**Figure 2:**
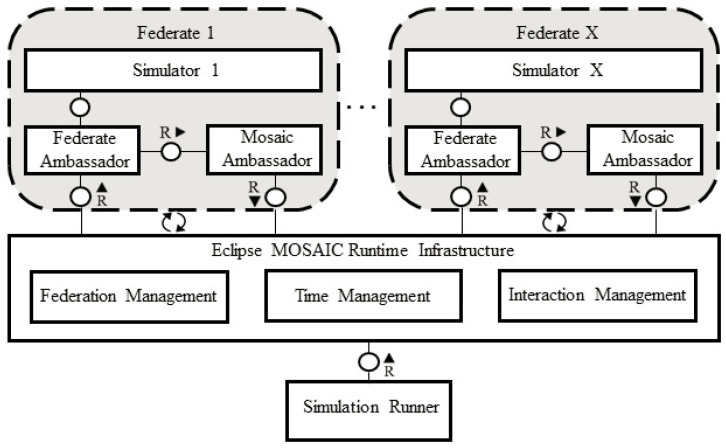
MOSAIC architecture.

A distributed architecture is used in Eclipse MOSAIC to facilitate the coupling of multiple simulators, called “federations”, to create a comprehensive simulation environment. This innovative approach allows the seamless integration of various simulators, each with its unique strengths and specializations, to model complex systems and scenarios. Federations, which can include simulators such as Eclipse SUMO (traffic simulator), NS-3 (network simulator) [39], or other custom simulators, are accompanied by “ambassadors” that serve as intermediaries between federations and the MOSAIC core. These ambassadors play a crucial role in facilitating communication and data exchange between federations and the MOSAIC core, converting each federation’s input and output data into a standardized format that can be understood by the environment. The MOSAIC core, which serves as the central nervous system of the simulation environment, manages the simulation process, coordinating the exchange of data between ambassadors, synchronizing time, and distributing results to other federations as needed. This ensures a highly synchronized and realistic simulation environment, where each federation can interact and influence the behavior of other federations in real time. The use of ambassadors and federations allows Eclipse MOSAIC to scale to large and complex simulations, making it an ideal tool for researchers and developers working on cutting-edge applications such as autonomous vehicles, smart cities, and vehicular cyber-physical systems [12]. This architecture is illustrated in Figure 2 [40], which shows the interactions between the MOSAIC core, ambassadors, and federations in a typical simulation scenario [12].

### 4.2. Simulation Scenarios

**Figure 3:**
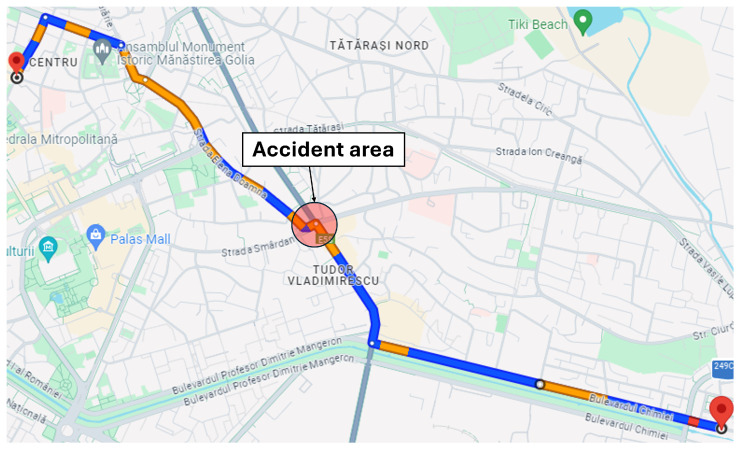
Scenario route.

**Figure 4:**
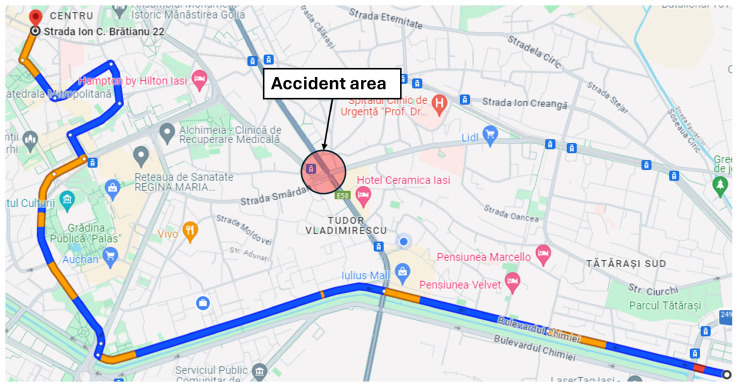
Alternative route for the single-accident scenario.

***Single-accident scenario:*** The first simulation scenario, implemented in the Eclipse MOSAIC framework, models a realistic urban traffic network in Iași (Figure 3). A fleet of vehicles is generated to traverse the route from Chimiei Boulevard to the Filarmonica area, representing peak-hour demand conditions. An unplanned incident is introduced in the Târgu Cucu region to evaluate network resilience under disruptive events. This scenario enables the assessment of adaptive traffic management strategies using key performance indicators such as congestion level, average travel time, and rerouting efficiency. It provides a controlled yet dynamic environment for testing V2X-enabled intelligent transportation systems and extracting insights applicable to real-world urban infrastructure design. Figure 3 depicts the affected route and accident site, while Figure 4 illustrates alternative routing options explored by the simulated vehicles.

**Figure 5:**
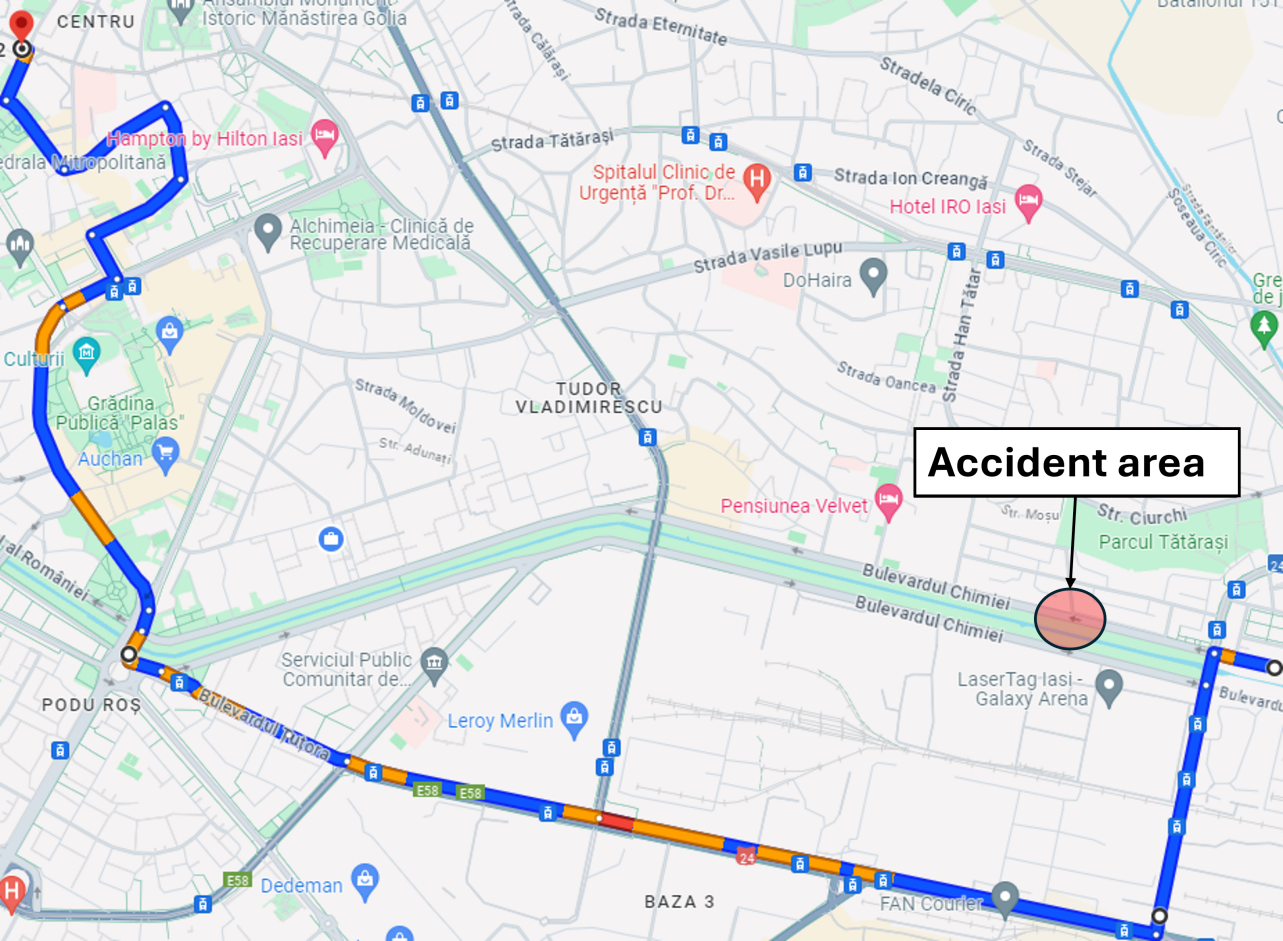
Alternative route for the dual-accident scenario.

***Dual-accident scenario:*** The second simulation scenario retains the same network parameters and origin–destination pairs as the initial case, ensuring consistent baseline conditions. In addition to the primary incident at Târgu Cucu, a secondary accident is introduced at coordinates (47.15230539775965, 27.61697250318553) on Chimiei Boulevard (Figure 5), creating a compound disruption within the network. To analyze the system’s response, affected vehicles are rerouted via Calea Chișinăului, enabling the evaluation of alternative path selection under multi-incident conditions. This scenario facilitates the assessment of intelligent transportation system performance in terms of congestion propagation, travel time variability, and rerouting efficiency when subjected to simultaneous network disturbances.

In the MOSAIC simulation, the accident event is parameterized to accurately reproduce the dynamics of a real traffic incident. The event is detected by a sensor defined as type “OBSTACLE” with a value of 1, indicating the presence of a physical blockage on the roadway. The location is set in the Târgu Cucu area, modeled as a circular zone centered at latitude 47.15886 and longitude 27.60108, with a 100-m radius defining the affected region. The event duration spans from 0 s to 3000 s, covering the entire simulation period to capture both immediate and sustained impacts on network performance. These configurations enable a controlled yet realistic representation of incident-induced disruptions, providing critical data for evaluating congestion patterns, vehicle behavior, and the effectiveness of adaptive traffic management strategies.

### 4.3. Vehicle Configuration

In the selected simulation scenario, vehicle behavior is defined via a JSON configuration specifying essential physical and dynamic parameters. The “Car” prototype is modeled with a length of 4.0 m, a minimum inter-vehicle spacing of 2.5 m, and a maximum speed of 70.0 m/s. Dynamic properties include an acceleration of 2.6 m/s^2^;, deceleration of 4.5 m/s^2^, a reaction time parameter (σ) of 1.0s, and a driver variability coefficient (τ) of 0.5. Vehicles are generated with a target flow of 2000 vehicles per hour (1.8 s headway), capped at 1000 units, and spawned at a constant rate with lane selection optimized using the “BEST” mode.

Traffic Management Centers (TMCs), such as “Road-Management”, are integrated into the network using the “RoadManagementApp” and are supported by strategically placed detectors in key locations (e.g., “Tudor_0”, “Chimiei_1”, “Palat”, “Chisinaului”) to capture real-time traffic flow data. Vehicles are also equipped with V2X-enabled applications, including “AccidentNotification”, “SumoTraciInteractionAppTimeLoss”, and “SumoTraciInteractionAppWaitingTime”, enabling interaction with the infrastructure and monitoring of critical performance metrics such as time loss and waiting time.

This detailed configuration allows the simulation to accurately reproduce complex urban traffic dynamics, facilitating rigorous analysis of vehicle behavior and supporting the evaluation of adaptive traffic management strategies under varying flow conditions.

### 4.4. Communication Method

In the proposed scenarios, the first incident is located in the Târgu Cucu area and the second on Chimiei Boulevard. Vehicles approaching these locations receive immediate warnings via V2X communication, enabling collision avoidance and proactive rerouting. Leveraging Eclipse MOSAIC’s real-time communication framework, vehicles can exchange critical safety information, reducing secondary accidents, mitigating congestion, and minimizing emergency response delays. For example, upon detecting an obstacle, a vehicle can broadcast warning messages to nearby units, prompting speed reduction or alternative route selection, thereby enhancing overall network resilience.

**Figure 6:**
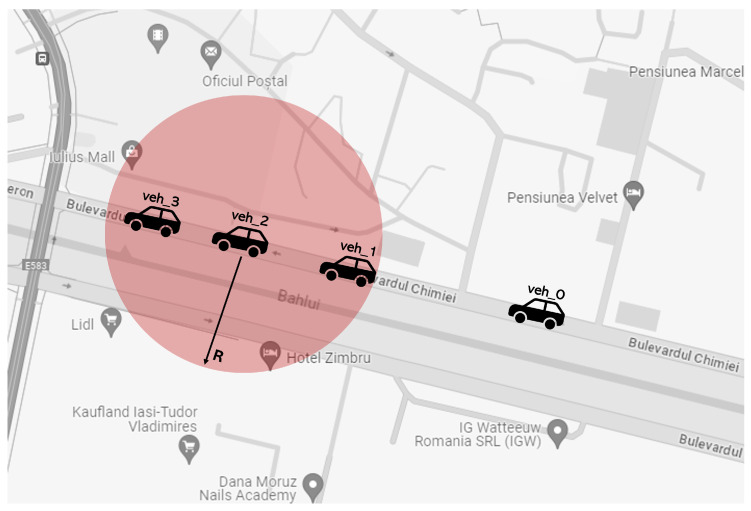
Illustration of cellular broadcast in a circular area.

A key enabler for such functionality is Cellular Geobroadcast, which disseminates messages to all devices within a defined geographic area without requiring individual addressing [41]. This mechanism, illustrated in Figure 6, uses existing cellular infrastructure to simultaneously alert all vehicles within a specified radius *R*, such as veh_2 transmitting to veh_1 and veh_3. The broadcast zone can span a single cell, a cluster of cells, or an entire monitoring area, offering scalable and targeted message distribution. In dense urban networks and highway environments, cellular geocasting ensures timely information delivery across the entire affected region, supporting adaptive traffic control and coordinated emergency response.

Deploying this capability requires integration between network operators, application developers, and municipal authorities to ensure robust infrastructure, secure message delivery, and seamless interoperability with intelligent transportation systems.

### 4.5. Communication Network

The vehicle-to-vehicle communication network in this study is configured to ensure high reliability and low-latency data exchange between traffic participants. At the global level, the network operates with uplink and downlink capacities of 28,000 kbps and 42,200 kbps, respectively, each maintaining a constant 100 ms delay to preserve synchronization across all vehicles.

To guarantee robust data delivery, uplink transmissions are configured with a 0.0% packet loss probability and allow up to two retransmissions, ensuring critical messages reach their destination even under transient errors. Downlink unicast channels adopt the same reliability parameters, while multi-cast transmission operates at 60% usable capacity under identical delay and loss constraints, enabling efficient dissemination of information to multiple vehicles simultaneously.

This configuration underscores the importance of minimizing latency and packet loss in vehicular networks, as precise synchronization and consistent data exchange are essential for cooperative maneuvers, congestion mitigation, and enhanced road safety. The resulting high-performance communication infrastructure forms a critical foundation for the deployment of advanced, V2X-enabled intelligent transportation systems.

### 4.6. Accident Notification

The *AccidentNotification* Java application, developed within the MOSAIC framework, manages incident detection and disseminates critical information to nearby vehicles using Vehicle-to-Everything (V2X) communication. Extending the *AbstractApplication* class and implementing *VehicleApplication* and *CommunicationApplication* interfaces, it dynamically activates either cellular or AdHoc communication modules depending on network availability.

Upon detecting a hazardous event, the application generates a Decentralized Environmental Notification Message (DENM) containing obstacle type, location, and severity. The message is transmitted via the active network interface, enabling surrounding vehicles to assess the event’s impact on their routes. If affected, the application invokes a navigation module that computes alternative paths using a cost-based routing algorithm, allowing vehicles to bypass the incident area. Visual indicators, such as color changes, reflect state transitions, including message reception and route adjustment.

By enabling real-time incident detection, message dissemination, and adaptive rerouting, *AccidentNotification* demonstrates the integration of V2X technologies and dynamic navigation modules for improving safety and maintaining traffic flow in complex urban scenarios. This approach supports rapid network-wide responses to disruptions and enhances the overall resilience of intelligent transportation systems.

### 4.7. Simulation Parameters and Scenario Selection

To ensure clarity and reproducibility, this subsection summarizes the key simulation parameters of the MOSAIC–SUMO framework and provides the rationale for selecting the study area and accident scenarios.

The main vehicle characteristics, accident configurations, simulation setup, and communication model are consolidated in Table 2. This structured overview complements the detailed descriptions in previous subsections by presenting all parameter values in a single reference, thereby facilitating comparison and reproducibility.

The city of Iași was chosen as the case study because it represents a typical mid-sized European urban center with a dense historical core and limited roadway expansion possibilities, comparable to cities such as Cluj-Napoca (Romania) and Kraków (Poland). Within this network, the Târgu Cucu intersection was selected for its role as a critical bottleneck frequently affected by congestion, while Chimiei Boulevard was included as a secondary disruption site given its function as a major arterial route connecting industrial and residential districts. These locations are representative of high-impact traffic hotspots and are therefore suitable for evaluating the resilience of intelligent transportation systems under both single- and dual-incident conditions.

## 5. Simulation Tests and Results

This section details the simulation scenarios with single- and dual-accidents and presents the illustrative results.

### 5.1. Single-Accident Simulation Scenario

**Figure 7:**
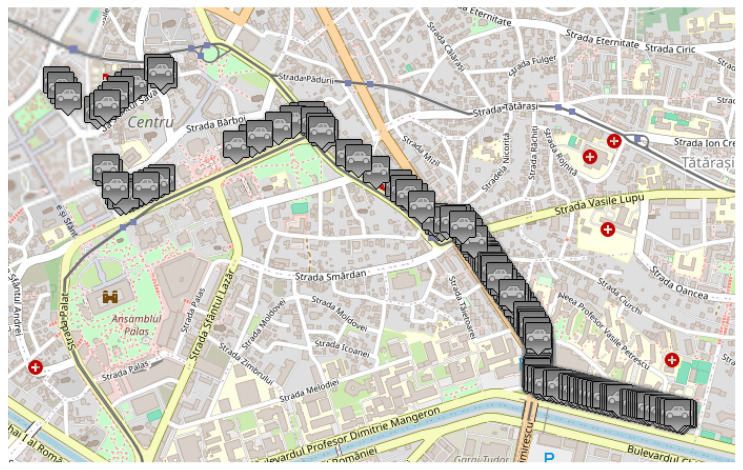
MOSAIC scenario with 0% equipped vehicle distribution. Grey color for a vehicle means that the vehicle does not have communication capabilities.

**Figure 8:**
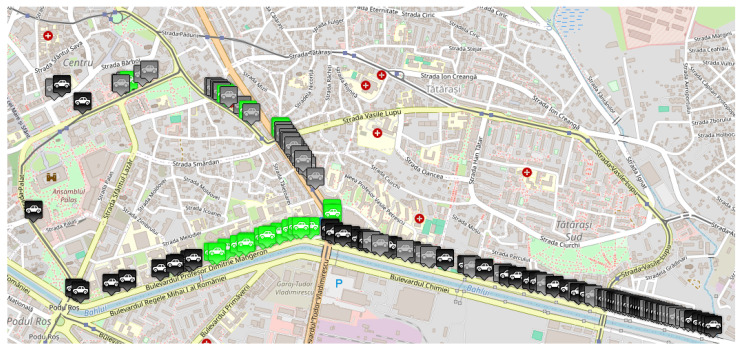
MOSAIC scenario with 50% equipped vehicle distribution. Grey color for a vehicle means that the vehicle does not have communication capabilities; black color for a vehicle means that the vehicle has communication capabilities; green color for a vehicle means that the vehicle has received information about the accident.

**Figure 9:**
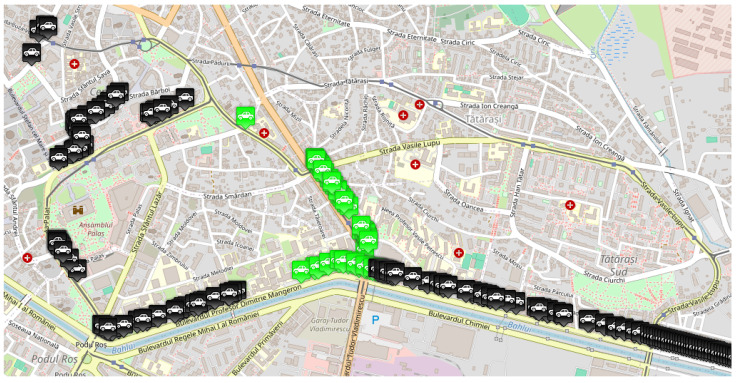
MOSAIC scenario with 100% equipped vehicle distribution. Grey color for a vehicle means that the vehicle does not have communication capabilities; black color for a vehicle means that the vehicle has communication capabilities; green color for a vehicle means that the vehicle has received information about the accident.

As previously mentioned, traffic data were collected by running the same scenario, imposing different densities for vehicles having equipment capable of sending and receiving messages when they encounter an obstacle on their route. Therefore, three traffic densities were considered for this study: 0%, 50%, and 100% of vehicles equipped with transceivers. In the MOSAIC simulation environment, these configurations were achieved by modifying the “weight” parameter of each vehicle type, thus establishing the probability distribution for the whole number of cars. These scenarios simulated various configurations, briefly described in what follows:

- The *base scenario* where no vehicle is equipped with such technology and therefore does not know the obstacles in advance, illustrated in Figure 7;
- An *intermediate scenario* shown in Figure 8 in which half of the vehicles are equipped;
- A *full scenario* in which all vehicles are equipped with this technology, illustrated in Figure 9.

Thus, by considering the base case and the alternative route options, a comprehensive assessment of the impact of V2V communication on traffic flow and route optimization can be performed. When vehicles detect an accident in the Târgu Cucu area, they will broadcast messages to other vehicles, propagating real-time information about the event. This cascade of communication will continue until vehicles approaching the intersection in the Iulius Mall area receive the alerts, allowing them to make timely decisions about their route.

### 5.2. Dual-Accident Simulation Scenario

**Figure 10:**
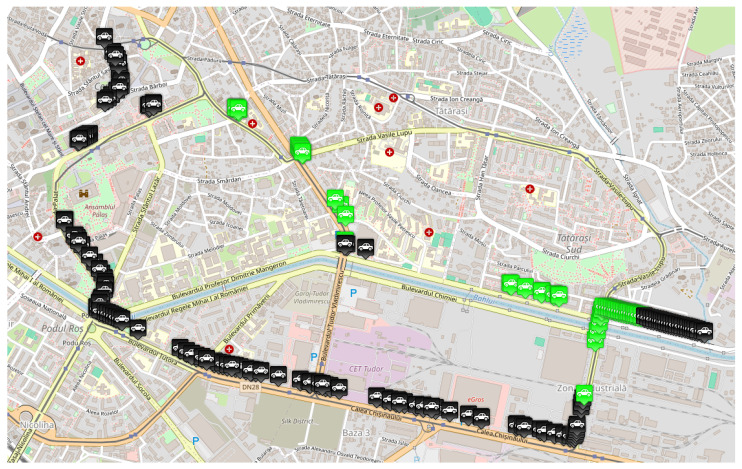
MOSAIC scenario including the second accident.Black color for a vehicle means that the vehicle has communication capabilities; green color for a vehicle means that the vehicle has received information about the accident.

Figure 10 presents a screenshot of the second scenario running in MOSAIC, highlighting how vehicles are redistributed along alternative routes—particularly via Calea Chișinăului to mitigate the effects of both incidents. This visualization is crucial for understanding the rerouting’s impact on traffic flow and for assessing the effectiveness of the implemented measures in managing multiple concurrent incidents.

### 5.3. Illustrative Results

The analysis focuses on several key performance indicators. First, the average vehicle speed along the alternative route is measured and compared against the baseline scenario without rerouting, as speed serves as a primary indicator of network fluidity and detour efficiency. Second, vehicle density variations on both the primary and alternative routes are examined to identify potential congestion build-up and evaluate network load distribution. Third, total travel time loss and waiting times at intersections are quantified and contrasted with the initial scenario to assess the effectiveness of the rerouting strategy under multi-incident conditions. Together, these metrics provide a detailed evaluation of the intelligent transportation system’s ability to manage simultaneous disruptions and offer insights into optimizing rerouting algorithms for improving traffic resilience in dense urban environments.

#### 5.3.1. Single-Accident Scenario

The following section presents experimental results for the simulation scenario described in Section 5.1, where 386 vehicles travel from Chimiei Boulevard to the Filarmonica area with an incident occurring in the Târgu Cucu zone. Analysis focuses on three critical segments of the route—Chimiei Boulevard (lanes 0 and 1), Tudor Vladimirescu Boulevard (lanes 0 and 1), and Palat Street—to evaluate the accident’s impact on traffic flow and congestion. Key performance indicators include average speed (m/s) and traffic density (veh/km), providing insight into the effectiveness of the simulation in replicating real-world traffic dynamics for intelligent transportation system design.

**Figure 11:**
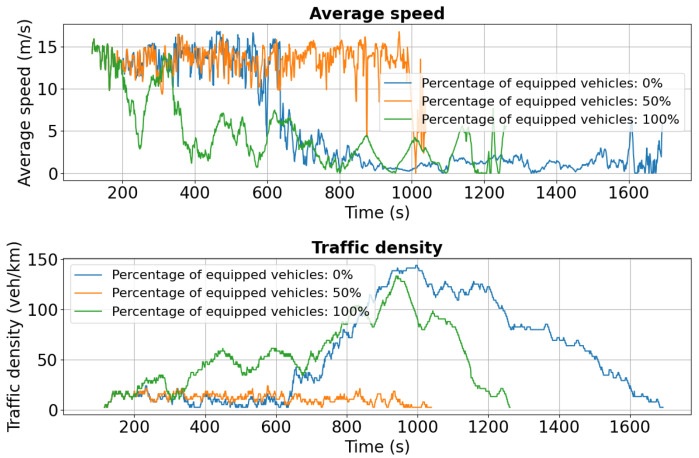
First scenario: Speed and density profiles on Chimiei Boulevard (lane 0) under different V2X penetration levels. Non-equipped vehicles (0%) experience a sharp drop to near standstill, while 50% and 100% equipped fleets maintain higher speeds through rerouting.

**Figure 12:**
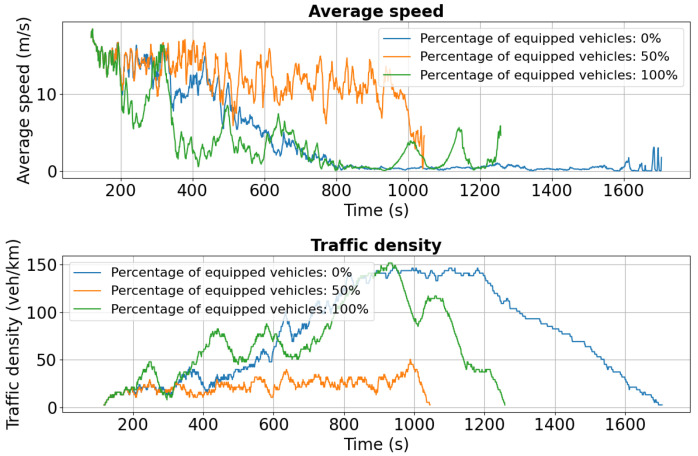
First scenario: Speed and density profiles on Chimiei Boulevard (lane 1). The unequipped case shows severe congestion with prolonged low speeds, while V2X-equipped scenarios maintain smoother flow.

For Chimiei Boulevard, Figure 11 and Figure 12 show speed and density profiles for both lanes. After approximately 800 s, unequipped vehicles (blue curve) experience a sharp decline in average speed to 0–2.5 m/s, while traffic density spikes to 140 veh/km. This pattern indicates severe congestion and extended idling periods, highlighting the significant influence of the Târgu Cucu accident on network performance and local traffic dynamics.

**Figure 13:**
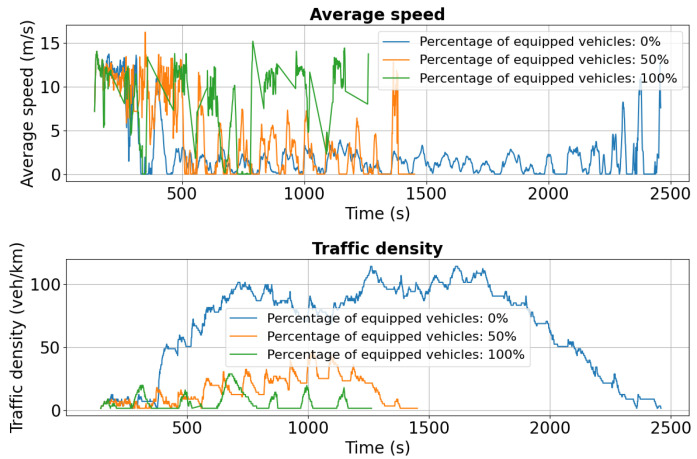
First scenario: Speed and density profiles on Tudor Vladimirescu Boulevard (lane 0). Traffic density in the 0% case surpasses 80 veh/km, while partial and full penetration reduce congestion by half.

**Figure 14:**
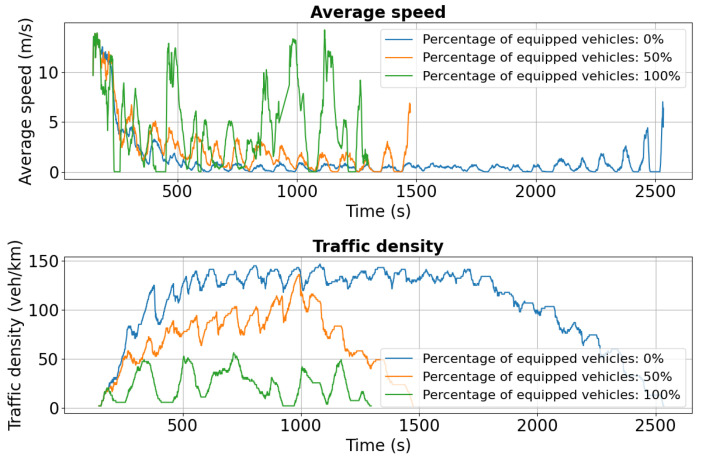
First scenario: Speed and density profiles on Tudor Vladimirescu Boulevard (lane 1). Vehicles without V2X remain near gridlock, while equipped vehicles benefit from rerouting and maintain stable flow.

Simulation results for Tudor Vladimirescu Boulevard, shown in Figure 13 and Figure 14, reveal distinct trends in speed and traffic density for both lanes. In Figure 13, the density of unequipped vehicles in lane 0 surpasses 80 veh/km after 500 s, indicating severe congestion, while equipped vehicles maintain significantly lower densities of 40 veh/km or less due to effective rerouting. This contrast underscores the impact of adaptive traffic management in mitigating bottlenecks and improving flow. Figure 14 further highlights these effects, with non-equipped vehicles in lane 1 exhibiting extremely low speeds (0–1 m/s), signaling heavy congestion and prolonged travel times, conditions that can exacerbate emissions and reduce overall network efficiency.

**Figure 15:**
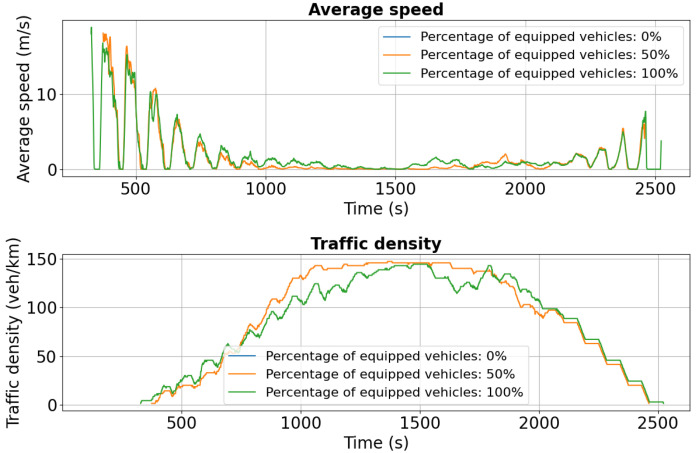
First scenario: Speed and density profiles on Palat Street. Despite full V2X penetration, severe congestion emerges due to capacity limits, illustrating that communication alone cannot overcome structural bottlenecks without complementary traffic management measures.

Figure 15, presenting the simulation results for Palat Street, reveals a counterintuitive outcome: the presence of only equipped vehicles results in severe congestion. Between 1000 s and 2000 s, traffic density peaks at approximately 140 veh/km, while average speed drops to nearly 0 m/s, indicating complete gridlock. This phenomenon underscores that even fully equipped fleets with advanced communication technologies cannot eliminate congestion when network capacity is exceeded, highlighting the need for complementary traffic management strategies and infrastructure optimization alongside V2X deployment.

#### 5.3.2. Dual-Accident Scenario

This subsection evaluates an additional scenario in which a second accident on Chimiei Boulevard is introduced alongside the existing incident in the Târgu Cucu area. The analysis uses the same performance indicators while incorporating a new area of interest—Calea Chișinăului—as part of the alternative routing strategy.

**Figure 16:**
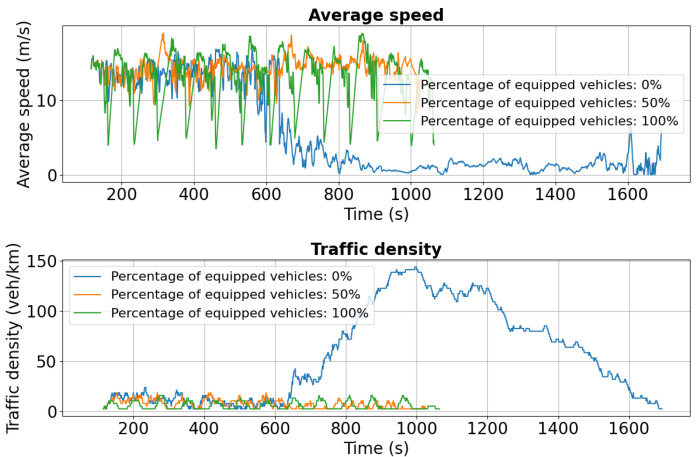
Second scenario: Speed and density profiles on Chimiei Boulevard (lane 0). The 0% scenario collapses to near zero speeds, while 50% and 100% penetration maintain average speeds around 10 m/s and 15 m/s, respectively.

As shown in Figure 16, initial average speeds are similar across all penetration levels (0%, 50%, 100%). However, after approximately 600 s, the 0% scenario exhibits a sharp decline to near 0 m/s, indicating severe congestion triggered by the added disruption. In contrast, the 50% case maintains a more stable average speed around 10 m/s, while the 100% scenario sustains the highest and most consistent performance, near 15 m/s.

**Figure 17:**
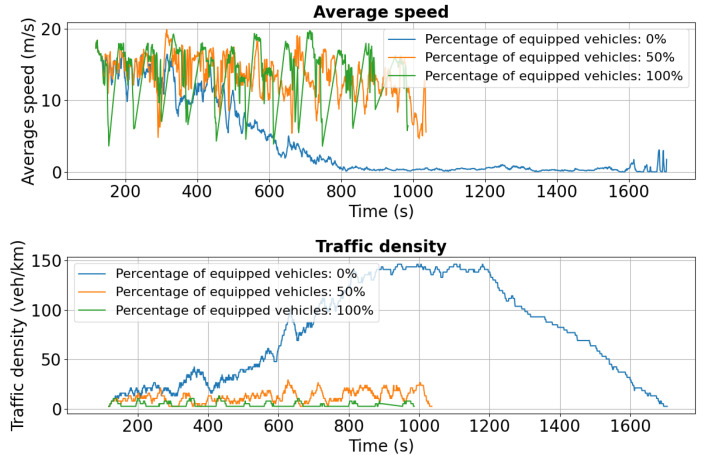
Second scenario: Speed and density profiles on Chimiei Boulevard (lane 1). V2X penetration significantly reduces the severity of congestion, with higher stability in the 50% and 100% cases compared to the baseline.

Traffic density trends reinforce these findings: in the 0% case, density peaks at 140 vehicles/km around 800 s, whereas 50% and 100% penetration maintain significantly lower levels at 60 and 40 vehicles/km, respectively, reflecting more uniform flow and reduced congestion. Figure 17 confirms the same pattern, with higher stability in both speed and density under partial and full V2X deployment.

These results demonstrate that intelligent vehicle systems substantially mitigate congestion in multi-incident scenarios, with performance improvements strongly correlated to V2X penetration rates.

**Figure 18:**
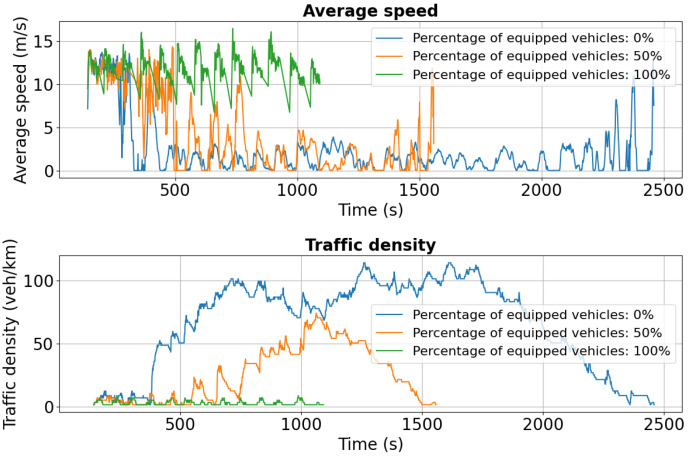
Second scenario: Speed and density profiles on Tudor Vladimirescu Boulevard (lane 0). The unequipped case exhibits rapid congestion build-up, whereas V2X-enabled vehicles sustain more balanced flows.

For Tudor Vladimirescu Boulevard, the dual-accident scenario exhibits similar patterns to other affected areas. As shown in Figure 18, the 0% penetration case experiences a marked reduction in average speed compared to the single-incident scenario, reflecting the network’s limited ability to absorb cascading disruptions. Vehicles equipped at 50% and 100% maintain higher average speeds but also show greater variability, indicating rerouting-induced fluctuations in traffic flow.

**Figure 19:**
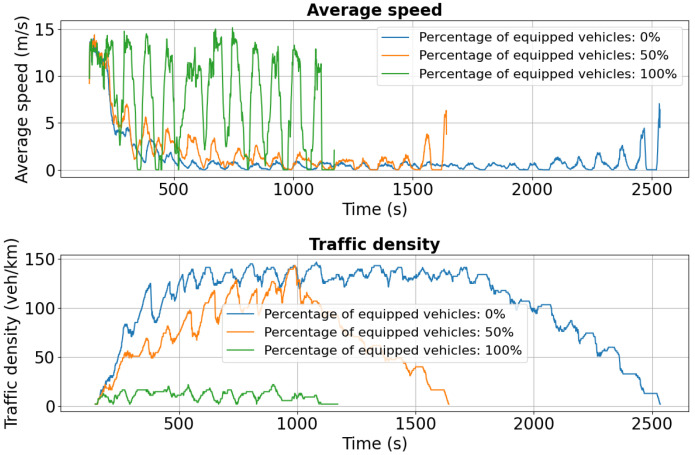
Second scenario: Speed and density profiles on Tudor Vladimirescu Boulevard (lane 1). Compared to the single-accident case, congestion intensifies across all penetration levels, but V2X-equipped fleets still demonstrate superior resilience.

Traffic density in lane 0 increases significantly for both the 0% and 50% cases, highlighting the formation of congestion clusters under partial or absent V2X deployment. Lane 1 (Figure 19) follows the same trend, with sharp speed reductions and density spikes across all penetration levels compared to the initial scenario (Figure 14).

These results underline the compounded impact of multiple incidents on network performance and reinforce the need for adaptive traffic management strategies, including real-time V2X-based rerouting and coordinated signal control, to sustain mobility under complex disruption scenarios.

**Figure 20:**
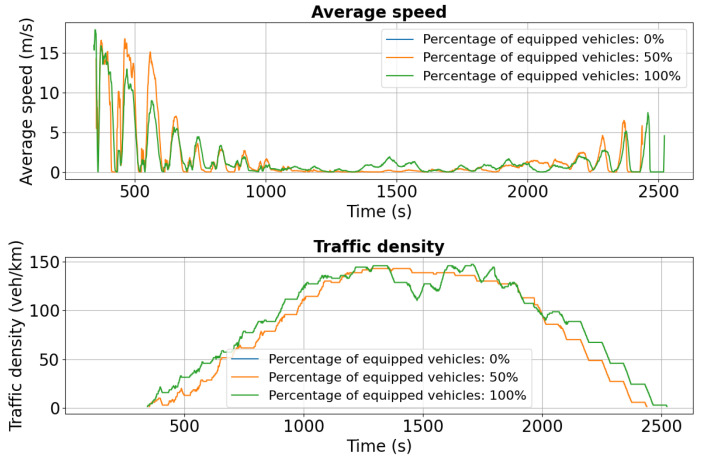
Second scenario: Speed and density profiles on Palat Street under dual-incident conditions. Rerouted vehicles increase density and reduce speed, showing how cascading effects overwhelm road capacity despite V2X support.

Figure 20 shows the simulation results for Palat Street under the dual-accident scenario, highlighting average speed and traffic density. Compared to the single-incident case, traffic conditions on this segment deteriorate significantly due to rerouted vehicles from Chimiei Boulevard and Târgu Cucu. Although Calea Chișinăului was designated as the primary detour, limited network capacity combined with high traffic volume resulted in severe congestion on Palat Street.

The influx of diverted vehicles reduced average speeds and increased density, emphasizing the need for more adaptive traffic management under cascading disruptions. Strategies such as dynamic signal optimization, advanced V2X-based rerouting, and better distribution of alternative paths can mitigate these effects. In the longer term, enhancing network capacity and strengthening public transport infrastructure are critical to improving resilience and maintaining flow during multi-incident scenarios.

**Figure 21:**
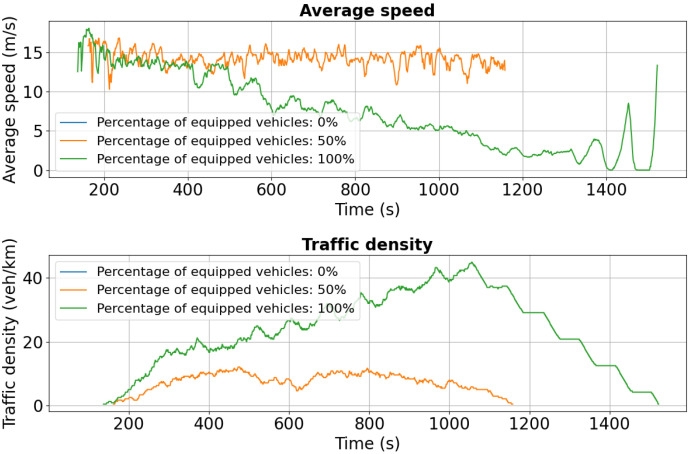
Second scenario: Speed and density profiles on Calea Chișinăului. Fully equipped vehicles sustain near-constant speeds (14 m/s) with higher throughput, while partial adoption shows moderate congestion and baseline conditions collapse under high demand.

The results for Calea Chișinăului (Figure 21) illustrate the impact of the second accident on Chimiei Boulevard and the effectiveness of intelligent transportation systems in maintaining traffic performance. For the 50% penetration scenario, the average speed remains around 13–14 m/s initially, gradually declining to 10 m/s after 600 s and 9 m/s after 1200 s, indicating moderate efficiency under disrupted conditions. In contrast, fully equipped (100%) vehicles sustain a nearly constant speed of 14 m/s for a longer duration, with only a slight decline over time, demonstrating superior resilience and flow stability. Traffic density metrics complement these findings: for 50% penetration, density rises from 10 vehicles/km to 25 vehicles/km after 600 s, signaling moderate congestion, while in the 100% scenario, density climbs more rapidly to 35 vehicles/km before stabilizing near 30 vehicles/km. This higher density, combined with maintained speeds, reflects increased network throughput and the ability of fully equipped fleets to adapt efficiently to changing traffic conditions.

## 6. Analysis and Discussion

The findings underline the importance of implementing intelligent transportation systems to manage and mitigate traffic congestion, especially during emergencies or multiple-accident scenarios. Equipped with advanced technology, vehicles can communicate effectively with each other and with road infrastructure, optimizing routes and significantly reducing waiting times and blockages.

The comparison between 50% and 100% equipped vehicles shows substantial gains in both average speed and traffic density. These results highlight the benefits of large-scale adoption of intelligent transportation technologies. These systems not only enhance traffic efficiency but also contribute to reducing pollution and fuel consumption by continuously optimizing vehicle flow.

In conclusion, the results indicate that fully equipped vehicles hold a significant advantage in maintaining constant speeds and managing traffic density, thereby contributing to a more efficient and sustainable transportation system.

### 6.1. Average Lost Time

An analysis of the information in Table 3, which presents a comparison of time loss data under different equipped vehicle distribution scenarios, reveals an interesting trend. Note that each simulation scenario (0%, 50%, and 100% V2X-equipped vehicles) was run five times using identical parameters to evaluate statistical variability. The results showed a consistent trend with the following average time losses (mean ± standard deviation).

#### 6.1.1. Scenario 1: Single-Accident

From Table 3, for the 0% penetration case, where no vehicles were equipped with transceiver technology, the average time loss was 921.84 s, representing the baseline congestion level. At 50% penetration, the average time loss increased slightly to 984.14 s, indicating that partial adoption does not yield significant efficiency gains and may even introduce network instability due to mixed traffic behavior. In contrast, full deployment (100%) reduced the average time loss to 758.18 s, demonstrating a substantial improvement in traffic flow and a marked decrease in congestion. These results emphasize that large-scale implementation of V2X technologies is critical to achieving meaningful reductions in travel time and enhancing overall traffic management

#### 6.1.2. Scenario 2: Dual-Accident

The data in Table 3 demonstrates the effect of a second accident on Chimiei Boulevard and the role of advanced vehicle technologies in mitigating its impact. In the unequipped scenario (0%), the average time loss reaches 921.84 s, indicating that traditional traffic management cannot effectively absorb multiple disruptions. At 50% penetration, the average time loss decreases sharply to 730.21 s, showing that even partial V2X adoption enables more efficient rerouting and congestion mitigation through vehicle-to-vehicle and vehicle-to-infrastructure communication. With full deployment (100%), the average time loss is 737.63 s, slightly higher than the single-accident case but still far below the unequipped scenario, highlighting the ability of intelligent transportation systems to maintain coordinated flow under cascading incidents. These results underscore three key points: advanced technologies substantially reduce delays, partial adoption already delivers measurable benefits, and large-scale implementation is essential for sustaining network resilience in complex traffic conditions.

### 6.2. Average Waiting Time

Table 4 provides data on the average waiting time of vehicles relative to the percentage of vehicles equipped with intelligent systems. As for the scenario with one accident, each simulation scenario (0%, 50%, and 100% V2X-equipped vehicles) was executed five times under identical parameters to assess statistical variability. The outcomes demonstrated a consistent pattern, with the following average time losses reported as mean ± standard deviation.

#### 6.2.1. Scenario 1: Single-Accident

The data in Table 4 illustrates the impact of intelligent vehicle penetration on average waiting times. With 0% equipped vehicles, the average waiting time is 9.22 s, increasing to 33.44 s at 50% penetration before decreasing to 27.47 s when all vehicles are equipped. This pattern highlights the nonlinear influence of V2X systems on traffic dynamics. The lowest waiting time in the unequipped scenario reflects conventional flow patterns without rerouting interventions. At 50% penetration, mixed traffic and partial rerouting introduce new congestion points—particularly evident on Palat Street—amplified further by the simulated accident in the Târgu Cucu area. With 100% equipped vehicles, enhanced communication and coordination reduce bottlenecks and mitigate delays, though waiting times remain above the unequipped case due to persistent congestion on heavily loaded corridors. These results underscore that the effectiveness of intelligent systems depends strongly on penetration rates and network conditions, with full deployment required to unlock their maximum potential.

#### 6.2.2. Scenario 2: Dual-Accident

The analysis of average waiting times in Scenario 2, which introduces a second accident on Chimiei Boulevard, provides important insights into traffic management under multiple disruptions. As shown in Table 4, waiting time is strongly influenced by the penetration level of intelligent systems: unequipped vehicles (0%) record an average of 9.22 s, partially equipped fleets (50%) increase to 33.78 s, while fully equipped fleets (100%) reduce the value to 26.96 s. Comparing these results with the initial scenario (9.22 s for 0%, 33.44 s for 50%, and 27.47 s for 100%) shows that the overall trend remains consistent despite the added disruption. The slightly lower waiting time for fully equipped vehicles in Scenario 2 (26.96 s vs. 27.47 s) suggests that V2X-enabled rerouting and coordination can maintain, and even marginally improve, network performance under cascading incidents. These results underline the effectiveness of large-scale deployment of intelligent vehicle technologies in minimizing delays and enhancing the resilience of urban traffic systems, contributing to more efficient and sustainable mobility.

### 6.3. Road Segment Exit Time

To complement the classical indicators of traffic performance, such as average speed, density, time loss, and waiting time, we also introduce the *road segment exit time* $T_{exit}$. This metric measures the time needed for all vehicles to clear a predefined road segment, thereby quantifying how efficiently traffic can recover after an incident. A smaller $T_{exit}$ indicates improved throughput and reduced congestion persistence.

Table 5 and Table 6 report the exit times for each analyzed road segment in Scenarios 1 and 2, under different V2X penetration rates (0%, 50%, and 100%). This comparison highlights the influence of communication-based rerouting on clearing congested segments after single- and dual-accident events.

As expected, the results confirm that higher V2X penetration levels reduce the clearance times of individual congested road sections, enabling faster recovery to free-flow conditions. In Scenario 1 (single-incident), both lanes of Chimiei Boulevard exhibit a reduction of more than 30% in exit time when V2X support is introduced, while Tudor Vladimirescu Boulevard shows improvements of up to 50%. A new traffic load appears on Palat Street at higher penetration rates, illustrating how rerouting redistributes congestion across the network. In Scenario 2 (dual-incident), the benefit of communication is even more pronounced on Tudor Vladimirescu Boulevard, where exit times fall by over 1200 s between the baseline and full penetration. Similarly, Chimiei Boulevard recovers nearly twice as fast, and additional diversion routes, such as Calea Chişinăului, are activated to balance demand. These results demonstrate that V2X-based rerouting not only accelerates clearance of primary blocked sections but also dynamically reallocates flows to alternative corridors, thereby enhancing network-wide resilience in both single- and multi-incident conditions.

### 6.4. Discussion

The simulation results demonstrate the transformative potential of large-scale V2X deployment in optimizing urban mobility. Fully equipping vehicles with advanced communication technologies reduced average travel time loss to 758.18 s, compared to 921.84 s in the baseline scenario without V2X. In contrast, partial deployment with 50% equipped vehicles yielded only modest improvements, with an average time loss of 984.14 s, underscoring the importance of widespread adoption to unlock the full benefits of cooperative traffic management. Traffic density and speed metrics further confirmed these trends: on Chimiei Boulevard, V2X-equipped fleets reduced density from 140 to approximately 20 vehicles per kilometer and increased average speed from near-zero to 10 m/s. On Tudor Vladimirescu Boulevard, the density of non-equipped vehicles in lane 0 exceeded 80 vehicles/km after 500 s, while the presence of V2X-enabled vehicles halved this value through efficient rerouting strategies.

In addition to classical performance indicators, the newly introduced road segment exit time metric provided further insight into network resilience. In the single-incident scenario, V2X penetration significantly reduced clearance times on the most affected arteries, with Chimiei and Tudor Vladimirescu Boulevards recovering considerably faster once cooperative rerouting was enabled. In the dual-incident scenario, the improvements were more localized, with noticeable reductions in exit times on Tudor Vladimirescu and Chimiei Boulevards, while detour routes such as Palat Street and Calea Chişinăului absorbed additional diverted flows. The smaller overall gains in this case reflect the saturation of alternative corridors: when multiple blockages occur simultaneously, rerouted vehicles compete for the same limited detour capacity, which constrains the benefits of communication. Even so, the results confirm that V2X-based rerouting accelerates recovery at the segment level and enhances resilience by distributing traffic more effectively during cascading disruptions.

Although calibrated for Iași, the methodology and results are transferable to other mid-sized European cities with comparable road networks and traffic patterns, such as Cluj-Napoca, Timișoara, or Kraków. The framework developed here offers a robust decision-support tool for urban mobility planners, capable of informing phased V2X deployment strategies and integration with existing traffic management systems. Future implementations could enhance this approach by incorporating live sensor data and adaptive feedback loops to bridge the gap between simulation-based planning and real-time operational control.

The results also provide actionable thresholds for policy and infrastructure investment. The sharp contrast between 50% and 100% penetration rates demonstrates that while partial deployment can yield measurable benefits, especially in managing simultaneous disruptions, full-scale adoption is critical to achieving network-wide efficiency. Municipal authorities can leverage these insights to prioritize V2X rollout along high-traffic corridors and critical intersections and to embed cooperative communication into emergency response protocols. The dual-incident scenario further highlights the role of V2X in maintaining resilience during cascading failures, emphasizing its strategic importance for future intelligent transportation systems.

### 6.5. State-of-the-Art Related Findings

In addition to using the 0% V2X penetration case as a baseline, it is important to contextualize our findings relative to traditional traffic management methods. Conventional approaches such as static signal timing, video-based adaptive signal control [8], or congestion pricing strategies [10] can partially mitigate delays by optimizing signal phases or discouraging peak-hour traffic. However, these methods remain centralized and infrastructure-dependent, with limited ability to adapt to sudden disruptions such as accidents. In contrast, V2X communication provides a decentralized and cooperative mechanism where vehicles exchange information in real time, enabling proactive rerouting and collision avoidance. For example, while congestion pricing may redistribute traffic demand, it does not provide incident-specific responses, and adaptive signals are often constrained to localized intersections rather than network-wide coordination. Our results demonstrate that V2X-enabled rerouting reduces average time loss by up to 18% and lowers density peaks by more than 70%, surpassing the improvements typically reported for static or semi-adaptive traffic control. This comparison highlights that V2X technologies complement, rather than replace, existing methods, providing faster and more resilient responses to cascading disruptions across urban networks.

## 7. Conclusions and Future Research Directions

This study demonstrated that integrating V2V and V2I technologies can substantially reduce congestion and optimize urban traffic flow, as validated using the MOSAIC simulation model. Analysis of key performance indicators, i.e., average speed, vehicle density, time loss, and waiting time, confirmed significant improvements under full V2X adoption, while partial deployment revealed efficiency gaps caused by limited V2V communication. The dual-incident scenario at Târgu Cucu and Chimiei Boulevard highlighted the limitations of conventional traffic management and the advantages of advanced technologies in enabling dynamic rerouting and maintaining flow under disruptive conditions. The proposed framework advances V2X-enabled ITS research by introducing a dual-incident simulation environment to capture cascading disruptions, quantifying the effects of different V2X penetration levels through statistically validated performance indicators, and integrating Eclipse MOSAIC with SUMO to bridge microscopic traffic dynamics with network-level communication modeling. Together, these contributions provide a transferable methodology for mid-sized European cities and deliver actionable insights for designing resilient urban mobility systems during the transition to full V2X deployment.

Future research will extend this work in three directions: (1) optimizing deployment strategies for advanced vehicle technologies across varied urban topologies and demand patterns; (2) integrating V2X systems with complementary ITS solutions, including adaptive signaling and dynamic lane management; and (3) evaluating the economic and environmental impacts of large-scale V2X adoption to inform sustainable urban transport policies. Collectively, these efforts will support the design of robust, efficient, and scalable intelligent transportation systems. Future work will also enhance the V2X framework with reinforcement learning for adaptive control, metaheuristics (genetic algorithms (GAs), particle swarm optimization (PSO)) for large-scale rerouting, and hybrid approaches combining IoT sensing with cloud/fog computing. These methods will strengthen prediction, resilience, and real-time optimization, bridging simulation results with practical traffic management solutions.

## Figures and Tables

**Table 1:** Comparison with related work on V2X-enabled adaptive traffic management.

Ref.	Simulator	Scenarios	Metrics	Comm. Tech	V2X Penetration
[35]	Veins	Intersection traffic	Overall traffic control performance	V2X, V2I	All vehicles capable of V2X communication
[36]	Artery V2X (SUMO + network)	Highway merging scenario	Effectiveness of V2X communications for safety and efficiency	V2X	Not specified
[37]	SUMO	Expressway weaving areas	Road capacity, vehicle delay time, network operational efficiency	Internet of Vehicles (IoV), C-V2X	Not specified
[17]	MOSAIC	One scenario	Density, speed (aim to enhance efficiency and safety)	V2X	0%, 50%, 100%
[21]	SUMO	Multiple traffic scenarios	Traffic conflicts (38% reduction), latency (99% reduction vs. DSRC)	C-V2X, DSRC	60% AV penetration

**Table 2:** Key simulation parameters used in the MOSAIC–SUMO framework.

Category	Parameter	Value/Description
Vehicle configuration	Vehicle prototype length	4.0 m
	Minimum inter-vehicle spacing	2.5 m
	Maximum speed	20.0 m/s
	Acceleration	2.6 m/s^2^
	Deceleration	4.5 m/s^2^
	Reaction time (σ)	1.0 s
	Driver variability coefficient (τ)	0.5
	Vehicle flow	2000 veh/hour (1.8 s headway)
	Fleet size	1000 vehicles max
Accident modeling	Location (Scenario 1)	Târgu Cucu area, Iași
	Location (Scenario 2)	Additional accident on Chimiei Boulevard (47.1523, 27.6170)
	Affected region	Circular zone, 100 m radius
	Event duration	0–3000 s (entire simulation)
	Detection method	OBSTACLE sensor, value = 1
Simulation setup	Penetration levels	0%, 50%, 100% equipped vehicles
	Simulation runs	5 repetitions per configuration
	Performance indicators	Average speed, traffic density, time loss, waiting time
Communication model	Protocol	Cellular Geobroadcast
	Uplink/downlink capacity	28,000/42,200 kbps
	Delay	100 ms constant
	Packet loss	0% uplink, ≤40% multi-cast
	Retransmissions	Up to 2 allowed

**Table 3:** Average time loss.

Distribution of Equipped Vehicles	Scenario 1	Scenario 2
0%	921.84 ± 14.5 s	1128.84 ± 16.7 s
50%	984.14 ± 11.2 s	730.21 ± 9.8 s
100%	758.18 ± 10.3 s	737.63 ± 10.1 s

**Table 4:** Average waiting time.

Distribution of Equipped Vehicles	Scenario 1	Scenario 2
0%	9.22 ± 0.14 s	11.31 ± 0.16 s
50%	33.44 ± 0.48 s	33.78 ± 0.49 s
100%	27.47 ± 0.35 s	26.96 ± 0.37 s

**Table 5:** Road segment exit time for Scenario 1 (single-accident).

Road Segment	0% V2X	50% V2X	100% V2X
Chimiei Boulevard lane 0	1600 s	950 s	1050 s
Chimiei Boulevard lane 1	1600 s	950 s	1050 s
Tudor Vladimirescu Boulevard lane 0	2350 s	1300 s	1150 s
Tudor Vladimirescu Boulevard lane 1	2350 s	1350 s	1200 s
Palat Street	-	2350 s	2450 s

**Table 6:** Road segment exit time for Scenario 2 (dual-accident).

Road Segment	0% V2X	50% V2X	100% V2X
Chimiei Boulevard lane 0	1620 s	920 s	950 s
Chimiei Boulevard lane 1	1620 s	950 s	870 s
Tudor Vladimirescu Boulevard lane 0	2350 s	1500 s	1050 s
Tudor Vladimirescu Boulevard lane 1	2350 s	1550 s	1080 s
Palat Street	-	2330 s	2420 s
Calea Chisinaului	-	1050 s	1370 s

## Data Availability

The original contributions presented in this study are included in the article. Further inquiries can be directed to the corresponding author.

## Abbreviations

The following abbreviations are used in this manuscript:

C-ITS	Cooperative Intelligent Transportation System
C-V2X	Cellular Vehicle-to-Everything
DENM	Decentralized Environmental Notification Message
DRL	Deep Reinforcement Learning
ITS	Intelligent Transportation System
SUMO	Simulation of Urban Mobility
TraCI	Traffic Control Interface
V2I	Vehicle-to-Infrastructure
V2N	Vehicle-to-Network
V2V	Vehicle-to-Vehicle
V2X	Vehicle-to-Everything
VLC	Visible Light Communication
